# Association of yield-related traits in founder genotypes and derivatives of common wheat (*Triticum aestivum* L.)

**DOI:** 10.1186/s12870-018-1234-4

**Published:** 2018-02-20

**Authors:** Jie Guo, Weiping Shi, Zheng Zhang, Jingye Cheng, Daizhen Sun, Jin Yu, Xinlei Li, Pingyi Guo, Chenyang Hao

**Affiliations:** 1 College of Agronomy, Shanxi Agricultural University, Taigu, Shanxi 030801 China; 2 Key Laboratory of Crop Gene Resources and Germplasm Enhancment, Ministry of Agriculture/The National Key Facility for Crop Gene Resources and Genetic Improvement/Institute of Crop Sciences, Chinese Academy of Agricultural Sciences, Beijing, 100081 China; 3 College of Agronomy, Yangzhou University, Yangzhou, Jiangsu 225009 China

**Keywords:** Founder parents, GWAS, iSelect SNP assays, Yield

## Abstract

**Background:**

Yield improvement is an ever-important objective of wheat breeding. Studying and understanding the phenotypes and genotypes of yield-related traits has potential for genetic improvement of crops.

**Results:**

The genotypes of 215 wheat cultivars including 11 founder parents and 106 derivatives were analyzed by the 9 K wheat SNP iSelect assay. A total of 4138 polymorphic single nucleotide polymorphism (SNP) loci were detected on 21 chromosomes, of which 3792 were mapped to single chromosome locations. All genotypes were phenotyped for six yield-related traits including plant height (PH), spike length (SL), spikelet number per spike (SNPS), kernel number per spike (KNPS), kernel weight per spike (KWPS), and thousand kernel weight (TKW) in six irrigated environments. Genome-wide association analysis detected 117 significant associations of 76 SNPs on 15 chromosomes with phenotypic explanation rates (*R*^*2*^) ranging from 2.03 to 12.76%. In comparing allelic variation between founder parents and their derivatives (106) and other cultivars (98) using the 76 associated SNPs, we found that the region 116.0–133.2 cM on chromosome 5A in founder parents and derivatives carried alleles positively influencing kernel weight per spike (KWPS), rarely found in other cultivars.

**Conclusion:**

The identified favorable alleles could mark important chromosome regions in derivatives that were inherited from founder parents. Our results unravel the genetic of yield in founder genotypes, and provide tools for marker-assisted selection for yield improvement.

**Electronic supplementary material:**

The online version of this article (10.1186/s12870-018-1234-4) contains supplementary material, which is available to authorized users.

## Background

Wheat, the most widely grown grain crop providing the food requirements of about 35% of the global population, generates the largest total harvest and is the most traded grain commodity [1–3]. Studying and understanding the phenotypes and genotypes of its agronomic traits may result in an improvement its yield stability.

Single nucleotide polymorphisms (SNP), as third-generation molecular markers, are superior in automated genotyping [4–6]. There are many reports on the use of high-density Illumina iSelect 90 K SNP chips in generating linkage maps [7–9]. For example, Gao et al. [7] built a genetic linkage map of hexaploid wheat that included 5536 polymorphic SNP markers covering a genetic length of 3609.4 cM using the 90 K iSelect SNP array. Jin et al. [9] identified 46,961 polymorphic SNPs in a 176-RIL population derived from a Gaocheng 8901/Zhoumai 16 cross using the 90 K and 660 K SNP arrays, and they produced a genetic map with a total length of 4121 cM and marker density of 0.09 cM/marker in bread wheat.

In addition to genetic mapping SNP markers have unique advantages for genome-wide association studies (GWAS) of yield-related traits in cereal crops, including rice [10], barley [11] and common wheat [12–15]. In particular, Yu et al. [10] detected genes linked to kernel type (*GS3*) and weight (*GW5*) associated with grain quality in rice by genome-wide SNP scanning and high-density genetic maps. Cormier et al. [12] investigated 28 nitrogen use-related traits in 240 European wheat varieties in a GWAS study, detecting 1010 SNPs significantly associated with nitrogen utilization. Sukumaran et al. [14] scanned the whole genomes of 287 wheat varieties using the Illumina iSelect 90 K SNP array and identified loci significantly associated with yield traits. Specifically, four, one, and five loci were associated with grain yield (chromosomes 3B, 5A, 5B, 6A), kernel weight (6A), and maturity (2B, 3B, 4B, 4D, 6A), respectively.

Analysis of the breeding history of many crop species revealed the presence and roles of founder parents. Molecular markers were used to analyze the contributions of the genetic bases of founder parents in improvement of barley [16], sugarcane [17], rice [18–20], and wheat [21, 22]. For example, Li et al. [19] and Tan et al. [20] built genetic maps of rice showing that quantitative trait loci (QTLs) of kernel number per spike, thousand-grain weight, and yield in the founder parent Minghui 63 were transmitted to the progenies over generations. By pedigree tracking of the founder parent Beijing 8, Li et al. [21] found that the frequencies of alleles unique to Beijing 8 varied from 0 to 0.96 in its 51 descendants, suggesting that some of them underwent rigorous artificial selection. Jiang et al. [22] confirmed that Ningmai 9 could serve as a founder parent and found some significant chromosome regions that might be used in wheat breeding.

In this study we genotyped 215 wheat cultivars using the iSelect 9 K SNP array, including 11 founder parents and 106 derivatives. Based on multi-environmental trial data we used GWAS to identify favorable alleles of yield-related traits through sequential generations of breeding. Favorable alleles identified in derivatives could be used to detect important chromosome regions inherited from the founder parents. This information might be used for marker-assisted selection (MAS) in wheat breeding.

## Results

### Phenotypic assessment

The average coefficients of variation for phenotypic traits in each environment ranged from 6.29 to 26.35%, indicating considerable phenotypic variation (Table 1). There were significant positive correlations between traits across environments (*P* < 0.01; Additional file 1: Table S1).

**Table 1:** Descriptive statistics of six phenotypic traits in different environments assessed in this study

	PH				SL				SNPS				KNPS				KWPS				TKW			
	Mean ± SD^a^	Min	Max	CV^b^ (%)	Mean ± SD^a^	Min	Max	CV^b^ (%)	Mean ± SD^a^	Min	Max	CV^b^ (%)	Mean ± SD^a^	Min	Max	CV^b^ (%)	Mean ± SD^a^	Min	Max	CV^b^ (%)	Mean ± SD^a^	Min	Max	CV^b^ (%)
09TA	92.15 ± 16.33	51.62	134.13	17.72	7.91 ± 1.07	5.22	10.71	13.53	20.88 ± 1.48	16.55	25.18	7.09	35.42 ± 7.05	19.78	64.93	19.90	1.33 ± 0.34	0.53	3.51	25.56	40.84 ± 5.17	26.33	61.19	12.66
09YL	91.21 ± 16.91	51.14	131.55	18.54	8.53 ± 1.25	4.92	12.41	14.65	20.35 ± 1.73	15.70	27.25	8.50	52.73 ± 6.39	38.80	70.63	12.12	2.20 ± 0.31	1.22	3.53	14.09	41.91 ± 5.03	27.43	61.50	12.00
09YZ	97.09 ± 14.34	55.83	133.33	14.77	9.87 ± 1.31	6.34	13.85	13.27	21.14 ± 1.37	17.57	24.63	6.48	54.69 ± 7.56	35.40	77.97	13.82	2.43 ± 0.42	1.27	3.83	17.28	38.53 ± 5.45	23.17	52.90	14.14
10TA	96.46 ± 17.42	52.31	142.19	18.06	8.46 ± 0.92	5.83	12.36	10.87	21.27 ± 1.66	12.74	27.22	7.80	36.58 ± 7.39	19.43	80.20	20.20	1.48 ± 0.39	0.40	3.60	26.35	39.87 ± 6.91	21.14	63.55	17.33
10YL	88.11 ± 13.54	50.27	127.40	15.37	8.80 ± 1.48	5.38	15.95	16.82	15.82 ± 2.47	11.07	23.27	15.61	52.58 ± 7.00	32.67	77.00	13.31	1.93 ± 0.37	0.96	3.09	19.17	37.71 ± 4.77	25.87	53.00	12.65
10YZ	97.62 ± 15.30	65.78	150.11	15.67	9.72 ± 1.34	6.49	17.07	13.79	19.32 ± 1.25	16.20	22.80	6.47	54.50 ± 9.61	33.67	81.73	17.63	2.05 ± 0.44	0.97	3.25	21.46	37.63 ± 5.03	22.43	52.07	13.37
Mean	93.65 ± 14.79	54.49	132.91	15.79	8.88 ± 1.05	5.91	11.97	11.82	19.72 ± 1.24	16.60	24.26	6.29	47.75 ± 6.03	33.51	72.60	12.63	1.90 ± 0.30	1.12	3.21	15.71	39.41 ± 4.63	26.57	53.60	11.75

^a^*SD* standard deviation

^b^*CV* coefficient of variation

*PH* plant height, *SL* spike length, *SNPS* spikelet number per spik, *KNPS* kernel number per spike, *KWPS* kernel weight per spike, *TKW* thousand kernel weight

The founder parents Funo, Bima 4, and Nanda 2419 and their derivatives over following generations were compared in terms of yield-related traits, including plant height (PH), spike length (SL), spikelet number per spike (SNPS), kernel number per spike (KNPS), kernel weight per spike (KWPS), and thousand kernel weight (TKW). PH gradually declined and TKW increased with advancing generations, while SL, SNPS, KNPS, and KWPS showed no significant changes. This indicated continuing selective pressure on PH and TKW during breeding (Additional file 2: Table S2).

### Allelic diversity and genetic structure

Genotyping of the 215 wheat cultivars using 9 K SNP array identified 4138 polymorphic SNPs, of which 3792 were mapped to single chromosome positions. Among them, 1795 were present in the A genome chromosomes, 1787 in the B genome, and only 210 in the D genome (Additional file 3: Table S3). Genetic diversity was analyzed using the 3792 SNPs. Gene diversity and polymorphism information content (PIC) ranged from 0.009 to 0.500 and from 0.009 to 0.375, with averages of 0.319 and 0.256, respectively. Major allele frequencies reached a maximum of 0.995, with an average of 0.762 (Additional file 3: Table S3), indicating that the germplasm was highly diverse.

**Fig. 1:**
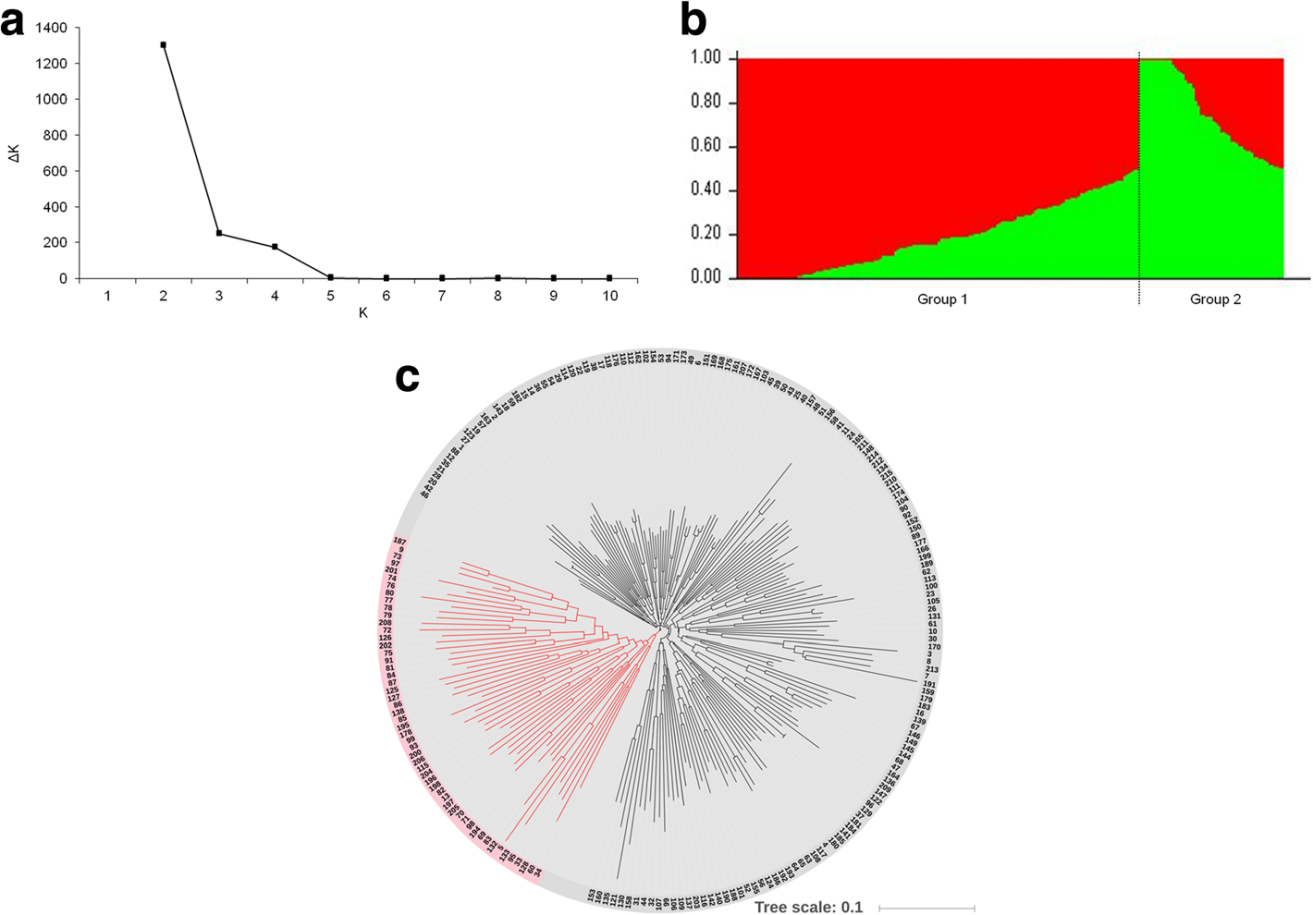
Population structure and neighbor-joining (NJ) tree of 215 cultivars based on 3792 unlinked SNP markers. (**a**) plot of ΔK against putative K ranging from 1 to 12; (**b**) stacked bar plot of ancestry relationships of 215 wheat cultivars; (**c**) NJ tree based on Nei’s distance. The two divergent groups are shown in black (162) and red (53), respectively

The number of subpopulation (K) was plotted against the ΔK calculated from the Structure, and the peak of the broken line graph was observed at K = 2 (Fig. 1a, b). The neighbor-joining method was used to classify 215 wheat cultivars based on Nei’s standard genetic distance [23], and they were divided into two groups (Fig. 1c). The first group (162) mainly consisted of Funo, Nanda 2419, and their derivatives, which mainly originated from Anhui, Henan, Hunan, Jiangsu, Shaanxi, and Sichuan provinces. The second group (53) mainly consisted of Bima 4 and its derivatives, which mainly originated from Beijing and Shandong. This further demonstrated that the population was basically divided into two subpopulations.

### Associations between yield-related traits and SNPs

**Fig. 2:**
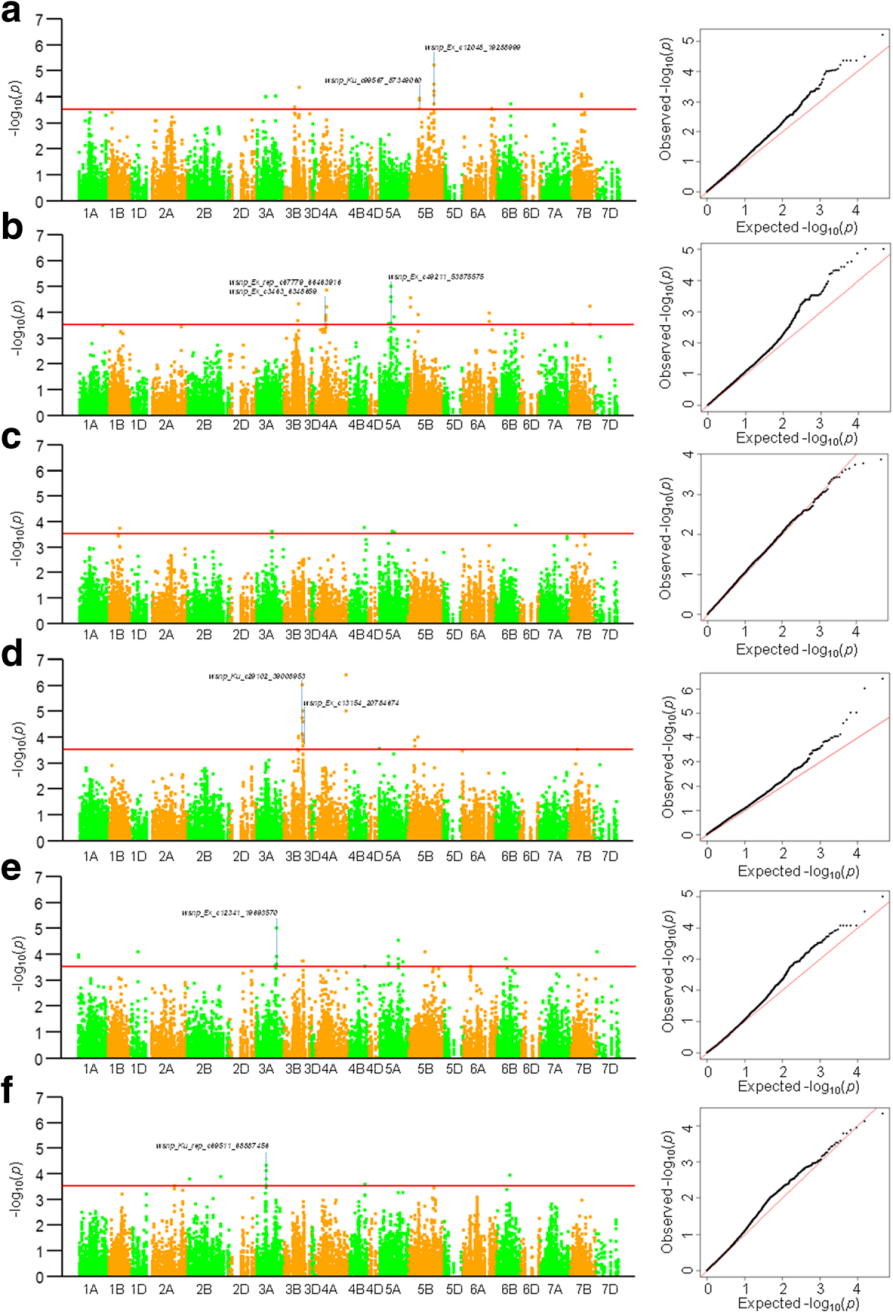
Manhattan and Q-Q plots of six phenotypic traits with 3792 genome-wide SNP markers shown as dot plots of compressed MLM at *P* < 3.06 × 10^− 4^. Red horizontal line corresponds to the threshold value for significant association. Green and orange colors separate different chromosomes. Significantly associated SNP markers are labeled with blue dotted lines. (**a**) SNPs *wsnp_Ex_c12048_19288999* and *wsnp_Ku_c99567_87349060* associated with PH were consistently detected in 5 and 3 environments, respectively; (**b**) SNPs *wsnp_Ex_rep_c67779_66463916*, *wsnp_Ex_c3463_6348659* and *wsnp_Ex_c49211_53875575* associated with SL were consistently detected in 4, 3 and 6 environments, respectively; (**c**) SNPs associated with SNPS were detected in less than two environments; (**d**) both *wsnp_Ku_c29102_39008953* and *wsnp_Ex_c13154_20784674* associated with KNPS were detected in 3 environments; (**e**) SNP *wsnp_Ex_c12341_19693570* associated with KWPS was detected in 3 environments; (**f**) SNP *wsnp_Ku_rep_c69511_68887456* associated with TKW was detected in 4 environments

Of the 3792 SNP markers, 3271 had a frequency higher than 0.05. Association analyses between the six yield-related traits and SNP markers showed that there were 117 significantly associated signals (*P* < 3.06 × 10^− 4^) among the 76 associated SNP loci, including 20, 35, 6, 23, 24, and 9 signals associated with PH, SL, SNPS, KNPS, KWPS, and TKW, respectively (Fig. 2). The phenotypic explanation rates (*R*^*2*^) ranged from 2.03 to 12.76%. The associated loci were located on 15 chromosomes (Table 2). Significant associations were found in two or more environments for 25 SNP loci; for example, *wsnp_Ex_c49211_53875575-5A* (SL) was significantly associated in all six environments, whereas others were significant in two to five environments (Table 2).

**Table 2:** One hundred and seventeen significant association signals (*P* < 3.06 × 10^− 4^) involving 76 associated SNP loci and six phenotypic traits

Trait	SNP Name	Chr.	Position	Alleles	Environment	*P* value	*R* ^*2*^
PH	*wsnp_Ra_c16846_25598885*	3A	54.99	A/G	09TA	1.00 × 10^−4^	7.90
	*wsnp_Ex_c13802_21639096*	3A	56.74	T/C	09YZ	1.02 × 10^− 4^	5.09
	*wsnp_BF292596A_Ta_1_1*	3A	119.09	T/C	10YZ	9.68 × 10^−5^	5.76
	*wsnp_JD_c8158_9193784*	3B	69.01	T/C	09TA	2.39 × 10^−4^	8.38
	*wsnp_CAP8_c4989_2410261*	3B	97.05	T/C	10YL	4.40 × 10^− 5^	5.00
	*wsnp_CAP8_c1419_836050*	3B	97.26	A/G	10TA	4.47 × 10^−5^	6.05
	*wsnp_Ex_c33463_41948471*	3B	97.72	A/G	10YL	4.45 × 10^−5^	7.41
	*wsnp_Ex_c12048_19288999*	5B	71.11	T/C	09TA	1.91 × 10^−4^	10.00
					09YZ	6.13 × 10^−5^	11.44
					09YL	8.95 × 10^−5^	9.89
					10YZ	3.27 × 10^−5^	3.33
					BLUP	6.31 × 10^−6^	10.79
	*wsnp_Ku_c99567_87349060*	5B	163.25	T/C	09YL	1.17 × 10^−4^	10.79
					09YZ	2.80 × 10^−4^	9.42
					10YZ	1.33 × 10^−4^	11.57
	*wsnp_Ex_c34597_42879693*	6A	180.19	T/C	09YL	2.97 × 10^−4^	10.39
					09YZ	2.91 × 10^−4^	10.82
	*wsnp_Ra_rep_c69821_67403173*	6B	82.68	T/C	10TA	1.81 × 10^−4^	3.48
	*wsnp_Ex_c15458_23737002*	7B	65.56	A/G	09TA	9.53 × 10^− 5^	11.17
					10TA	8.40 × 10^−5^	10.89
SL	*wsnp_JD_c6974_8084752*	3B	95.50	A/G	09TA	2.17 × 10^−4^	3.90
	*wsnp_CAP8_c4989_2410261*	3B	97.05	T/C	10TA	4.85 × 10^− 5^	3.02
	*wsnp_CAP8_c1419_836050*	3B	97.26	A/G	10YL	4.85 × 10^−5^	7.02
					BLUP	4.90 × 10^−5^	6.01
	*wsnp_Ex_c33463_41948471*	3B	97.72	A/G	09YZ	4.85 × 10^−5^	3.02
	*wsnp_Ex_c1563_2987002*	4A	136.22	A/G	10YL	6.13 × 10^− 5^	8.13
					BLUP	1.35 × 10^−5^	9.05
	*wsnp_Ex_c28092_37240192*	4A	140.47	A/G	10TA	2.03 × 10^− 4^	2.78
	*wsnp_Ex_rep_c67779_66463916*	4A	141.36	A/C	09YL	3.00 × 10^−4^	10.60
					09YZ	2.99 × 10^−4^	11.61
					10YL	1.37 × 10^− 4^	9.72
					BLUP	1.73 × 10^−4^	9.90
	*wsnp_Ex_c2266_4247520*	4A	141.80	A/G	10YL	2.99 × 10^−4^	10.61
					BLUP	3.00 × 10^−4^	8.60
	*wsnp_Ex_c12_21212*	4A	143.00	T/G	09YZ	3.00 × 10^− 4^	2.60
	*wsnp_Ex_c3463_6348659*	4A	144.11	A/G	10TA	2.99 × 10^−4^	7.61
					10YZ	3.00 × 10^− 4^	8.46
					10YL	2.29 × 10^−4^	9.02
	*wsnp_Ex_c27298_36506245*	5A	102.41	A/G	BLUP	1.54 × 10^−4^	3.89
	*wsnp_Ex_c49211_53875575*	5A	108.64	T/G	09TA	1.01 × 10^−5^	7.28
					09YZ	2.67 × 10^− 4^	8.24
					09YL	3.70 × 10^−5^	4.89
					10TA	3.79 × 10^−5^	6.89
					10YZ	1.11 × 10^−5^	5.08
					10YL	2.66 × 10^−4^	5.04
	*wsnp_Ex_c10127_16635328*	5A	108.92	T/C	09TA	2.54 × 10^−5^	2.34
	*wsnp_Ex_c19647_28632894*	5A	123.92	A/G	10YZ	2.80 × 10^−4^	2.64
	*wsnp_Ex_c1630_3105100*	5B	161.77	A/G	10YL	1.28 × 10^−4^	4.06
	*wsnp_Ex_c2459_4591695*	5B	212.53	A/G	10YL	2.75 × 10^−5^	2.13
					BLUP	6.45 × 10^−5^	2.25
	*wsnp_Ex_c8510_14306239*	6A	175.39	A/G	10YL	1.10 × 10^−4^	2.70
					BLUP	2.29 × 10^−4^	2.97
	*wsnp_Ex_c46061_51675853*	7B	25.00	A/G	10YZ	2.86 × 10^−4^	10.54
	*wsnp_Ex_rep_c101269_86664147*	7B	132.23	A/G	09YL	2.94 × 10^−4^	8.39
					10YZ	5.89 × 10^−5^	10.25
SNPS	*wsnp_BM140362B_Ta_1_1*	1B	76.37	A/G	10TA	1.90 × 10^−4^	2.80
	*wsnp_Ku_rep_c68484_67499824*	3A	99.60	T/C	09YZ	2.46 × 10^−4^	8.19
	*wsnp_Ex_rep_c67136_65617520*	4B	108.15	T/C	09YZ	1.75 × 10^−4^	5.15
	*wsnp_Ku_c21275_31007309*	5A	85.17	A/C	09YL	2.83 × 10^−4^	4.49
	*wsnp_RFL_Contig3939_4369467*	5A	94.73	T/C	09YL	2.38 × 10^− 4^	4.84
	*wsnp_Ex_c7713_13153321*	6B	127.53	A/C	09TA	1.41 × 10^− 4^	11.88
KNPS	*wsnp_JD_c6974_8084752*	3B	95.50	A/G	10TA	3.03 × 10^−4^	2.77
	*wsnp_Ex_c6129_10723019*	3B	97.26	T/C	09YZ	9.11 × 10^−5^	8.86
					10YZ	1.06 × 10^− 4^	7.79
	*wsnp_Ku_c29102_39008953*	3B	123.29	A/C	09TA	1.88 × 10^−5^	7.12
					09YL	7.46 × 10^−5^	12.61
					BLUP	9.61 × 10^−7^	11.49
	*wsnp_Ku_c31407_41142340*	3B	125.61	A/G	10YL	1.27 × 10^− 4^	8.77
	*wsnp_Ex_c11837_18996495*	3B	126.24	T/G	09YL	2.28 × 10^−4^	10.55
					BLUP	1.56 × 10^− 4^	8.69
	*wsnp_Ex_c12781_20280445*	3B	126.61	A/G	10YZ	1.43 × 10^−4^	9.05
					10YL	1.40 × 10^−4^	8.90
	*wsnp_Ex_c13154_20784674*	3B	127.87	T/C	09TA	8.85 × 10^−5^	7.86
					09YL	2.19 × 10^− 4^	11.25
					BLUP	9.62 × 10^−6^	11.53
	*wsnp_Ex_c13154_20785032*	3B	127.87	A/G	09TA	1.51 × 10^−4^	7.41
					BLUP	2.65 × 10^−5^	10.59
	*wsnp_Ex_c13953_21831752*	4A	13.89	T/C	09YZ	3.95 × 10^−7^	5.64
					10TA	9.75 × 10^−6^	6.42
	*wsnp_Ex_c16551_25060833*	5A	190.95	T/C	09TA	2.85 × 10^−4^	6.09
	*wsnp_Ex_c1630_3105100*	5B	161.77	A/G	10TA	1.04 × 10^−4^	2.03
	*wsnp_Ex_c1302_2489542*	5B	184.33	T/G	10YZ	2.35 × 10^−4^	2.72
	*wsnp_Ku_c56917_60245833*	5B	185.06	T/C	10YZ	1.31 × 10^− 4^	2.84
	*wsnp_Ku_c19037_28455905*	7B	57.38	A/G	09YZ	3.02 × 10^−4^	5.98
KWPS	*wsnp_Ex_rep_c106111_90308719*	1A	12.73	T/G	09YL	1.37 × 10^−4^	10.81
					BLUP	1.11 × 10^− 4^	10.78
	*wsnp_Ex_c1130_2166731*	1D	54.49	A/G	BLUP	8.41 × 10^−5^	2.78
	*wsnp_Ex_rep_c69919_68881108*	3A	117.88	A/G	10TA	3.02 × 10^−4^	2.74
	*wsnp_Ex_rep_c104327_89077792*	3A	118.07	A/G	09YZ	3.02 × 10^−4^	2.74
	*wsnp_Ra_c19079_28210937*	3A	123.35	A/C	09YL	2.91 × 10^−4^	2.97
	*wsnp_Ex_c12341_19693570*	3A	127.51	T/C	09TA	2.53 × 10^−4^	12.44
					10TA	1.00 × 10^−5^	10.36
					10YZ	1.21 × 10^−4^	11.94
	*wsnp_Ku_c29102_39008953*	3B	123.29	A/C	09YZ	1.93 × 10^−4^	3.15
	*wsnp_Ex_c13154_20785032*	3B	127.87	A/G	09TA	1.85 × 10^−4^	3.66
	*wsnp_Ex_rep_c67136_65617520*	4B	108.15	T/C	09YZ	2.98 × 10^−4^	10.43
	*wsnp_JD_rep_c49046_33288885*	5A	116.00	T/C	09YZ	1.52 × 10^−4^	10.51
					BLUP	2.97 × 10^−5^	11.94
	*wsnp_Ku_c14275_22535693*	5A	116.57	A/G	10TA	2.53 × 10^−4^	2.32
	*wsnp_Ku_c14275_22535576*	5A	116.83	T/C	10YZ	1.94 × 10^−4^	2.84
	*wsnp_Ex_c43578_49857984*	5A	130.98	T/C	09YL	2.91 × 10^−4^	12.48
					BLUP	1.24 × 10^−4^	12.76
	*wsnp_Ex_rep_c101757_87064771*	5A	133.01	T/C	09YZ	2.24 × 10^−4^	8.30
	*wsnp_Ex_rep_c101757_87065169*	5A	133.17	T/C	09TA	2.28 × 10^−4^	7.18
	*wsnp_Ex_c57667_59284398*	5B	125.89	T/C	09TA	8.37 × 10^−5^	5.19
	*wsnp_Ex_c1050_2009301*	6A	45.73	T/G	10TA	3.03 × 10^−4^	4.27
	*wsnp_Ex_c19467_28423197*	6B	50.70	A/G	09TA	1.56 × 10^−4^	4.80
	*wsnp_Ex_c65899_64135487*	7D	1.12	A/G	09YL	8.35 × 10^−5^	3.10
TKW	*wsnp_Ex_c3685_6723631*	2A	9.91	A/G	BLUP	1.65 × 10^−4^	4.76
	*wsnp_BE406351A_Ta_2_2*	2A	112.62	T/C	09TA	3.00 × 10^−4^	8.08
	*wsnp_RFL_Contig2914_2757372*	2B	211.84	A/G	10YL	1.35 × 10^−4^	8.27
	*wsnp_Ku_rep_c69511_68887456*	3A	59.73	T/C	09TA	1.68 × 10^−4^	11.41
					09YL	4.73 × 10^−5^	11.61
					09YZ	2.96 × 10^−4^	10.47
					BLUP	7.72 × 10^−5^	10.04
	*wsnp_CAP12_c4769_2174195*	4B	106.45	T/C	10TA	2.62 × 10^−4^	2.14
	*wsnp_BF291478B_Ta_2_1*	6B	80.00	T/C	09TA	1.61 × 10^−4^	4.65

### Phenotypic effects of yield-related alleles

The phenotypic effects of alleles were further analyzed (Table 3). Favorable alleles with larger genetic effects on PH, SL, SNPS, KNPS, KWPS and TKW were *wsnp_Ku_c99567_87349060-5B*_*CC*_ (reduction of PH by 8.82 cm in 09YL, 6.91 cm in 09YZ, and 5.90 cm in 10YZ), *wsnp_Ex_c1630_3105100-5B*_*AA*_ (1.34 cm in 10YL), *wsnp_Ex_c7713_13153321-6B*_*CC*_ (1.48 cm in 09YL); *wsnp_Ex_c13953_21831752-4A*_*CC*_ (increases in KNPS by 4.27 in 09YZ and 3.45 in 10YL); *wsnp_Ex_c19467_28423197-6B*_*AA*_ (increases in TKW by 0.26 g in 09YL); and *wsnp_Ku_rep_c69511_68887456-3A*_*TT*_ (increases in TKW by 1.41 g in 09TA, 1.01 g in 09YL, 1.48 g in 09YZ, and 1.33 g in BLUP), respectively. The frequencies of these alleles at associated loci ranged from 6.05 to 97.21%, and exceeded 50% for 64 alleles, indicating strong selection on those alleles in breeding.

**Table 3:** Favored alleles and genetic effects of 76 SNP loci significantly (*P* < 3.06 × 10^− 4^) associated with six phenotypic traits

Trait	SNP Name	Chr.	Position	Favored allele	Freq. (%)	Allele effect						
						09TA	09YL	09YZ	10TA	10YL	10YZ	BLUP
PH	*wsnp_Ra_c16846_25598885*	3A	54.99	GG	93.56	−1.50^*^						
	*wsnp_Ex_c13802_21639096*	3A	56.74	TT	93.56			−1.57^*^				
	*wsnp_BF292596A_Ta_1_1*	3A	119.09	CC	74.29						−1.49^*^	
	*wsnp_JD_c8158_9193784*	3B	69.01	CC	82.52	−2.69^*^						
	*wsnp_CAP8_c4989_2410261*	3B	97.05	CC	94.37					−1.10^*^		
	*wsnp_CAP8_c1419_836050*	3B	97.26	GG	94.37				−1.24^*^			
	*wsnp_Ex_c33463_41948471*	3B	97.72	GG	94.37					−1.21^*^		
	*wsnp_Ex_c12048_19288999*	5B	71.11	TT	85.78	−4.93^**^	−1.68^*^	− 1.87^*^			−1.53^*^	− 1.36^*^
	*wsnp_Ku_c99567_87349060*	5B	163.25	CC	90.73		−8.82^**^	−6.91^**^			−5.90^**^	
	*wsnp_Ex_c34597_42879693*	6A	180.19	TT	79.02		−6.88^**^	−6.07^**^				
	*wsnp_Ra_rep_c69821_67403173*	6B	82.68	TT	43.28				−3.06^*^			
	*wsnp_Ex_c15458_23737002*	7B	65.56	GG	61.43	−3.46^**^			−3.32^*^			
SL	*wsnp_JD_c6974_8084752*	3B	95.50	GG	93.49	0.80^*^						
	*wsnp_CAP8_c4989_2410261*	3B	97.05	CC	94.37				1.05^*^			
	*wsnp_CAP8_c1419_836050*	3B	97.26	GG	94.37					1.10^**^		1.09^**^
	*wsnp_Ex_c33463_41948471*	3B	97.72	GG	94.37			0.76^*^				
	*wsnp_Ex_c1563_2987002*	4A	136.22	GG	93.95					0.64^*^		0.72^*^
	*wsnp_Ex_c28092_37240192*	4A	140.47	GG	95.10				0.58^*^			
	*wsnp_Ex_rep_c67779_66463916*	4A	141.36	CC	94.84		0.40^*^	0.37^*^		0.49^*^		0.62^*^
	*wsnp_Ex_c2266_4247520*	4A	141.80	AA	94.81					0.38^*^		0.49^*^
	*wsnp_Ex_c12_21212*	4A	143.00	TT	94.84			0.49^*^				
	*wsnp_Ex_c3463_6348659*	4A	144.11	GG	94.81				0.48^*^	0.45^*^	0.60^*^	
	*wsnp_Ex_c13942_21820758*	5A	102.41	GG	91.63							0.89^*^
	*wsnp_Ex_c49211_53875575*	5A	108.64	GG	60.47	0.32^**^	0.42^**^	0.30^**^	0.27^**^	0.62^**^	0.52^**^	
	*wsnp_Ex_c10127_16635328*	5A	108.92	TT	64.19	0.18^*^						
	*wsnp_Ex_c19647_28632894*	5A	123.92	GG	53.49						0.48^*^	
	*wsnp_Ex_c1630_3105100*	5B	161.77	AA	94.81					1.34^**^		
	*wsnp_Ex_c2459_4591695*	5B	212.53	AA	94.88					0.97^**^		0.68^**^
	*wsnp_Ex_c8510_14306239*	6A	175.39	AA	94.39					1.02^**^		0.74^**^
	*wsnp_Ex_c46061_51675853*	7B	25.00	GG	19.34						0.89^*^	
	*wsnp_Ex_rep_c101269_86664147*	7B	132.23	AA	23.27		0.57^**^				0.78^**^	
SNPS	*wsnp_BM140362B_Ta_1_1*	1B	76.37	AA	93.84				1.08^*^			
	*wsnp_Ku_rep_c68484_67499824*	3A	99.60	TT	66.67			0.46^*^				
	*wsnp_Ex_rep_c67136_65617520*	4B	108.15	CC	87.25			0.73^*^				
	*wsnp_Ku_c21275_31007309*	5A	85.17	CC	36.28		0.45^*^					
	*wsnp_RFL_Contig3939_4369467*	5A	94.73	CC	88.37		1.08^**^					
	*wsnp_Ex_c7713_13153321*	6B	127.53	CC	89.47	1.48^**^						
KNPS	*wsnp_JD_c6974_8084752*	3B	95.50	GG	93.49				2.79^*^			
	*wsnp_Ex_c6129_10723019*	3B	97.26	CC	91.59			2.71^**^			2.50^**^	
	*wsnp_Ku_c29102_39008953*	3B	123.29	AA	74.04	3.18^**^	3.84^**^					3.45^**^
	*wsnp_Ku_c31407_41142340*	3B	125.61	AA	76.64					1.23^*^		
	*wsnp_Ex_c11837_18996495*	3B	126.24	GG	77.25		3.97^**^					3.27^**^
	*wsnp_Ex_c12781_20280445*	3B	126.61	GG	76.64					3.25^**^	3.27^**^	
	*wsnp_Ex_c13154_20784674*	3B	127.87	CC	76.92	3.59^**^	4.18^**^					3.73^**^
	*wsnp_Ex_c13154_20785032*	3B	127.87	AA	76.56	3.44^**^						3.55^**^
	*wsnp_Ex_c13953_21831752*	4A	13.89	CC	81.43			4.27^**^	3.45^**^			
	*wsnp_Ex_c16551_25060833*	5A	190.95	CC	19.53	2.01^*^						
	*wsnp_Ex_c1630_3105100*	5B	161.77	AA	94.81				2.49^*^			
	*wsnp_Ex_c1302_2489542*	5B	184.33	TT	89.37						2.62^*^	
	*wsnp_Ku_c56917_60245833*	5B	185.06	CC	89.47						2.74^*^	
	*wsnp_Ku_c19037_28455905*	7B	57.38	GG	80.90			0.88^*^				
KWPS	*wsnp_Ex_rep_c106111_90308719*	1A	12.73	TT	6.10		0.11^*^					0.10^*^
	*wsnp_Ex_c1130_2166731*	1D	54.49	GG	6.05							0.20^*^
	*wsnp_Ex_rep_c69919_68881108*	3A	117.88	AA	70.48				0.03^*^			
	*wsnp_Ex_rep_c104327_89077792*	3A	118.07	AA	70.48			0.03^*^				
	*wsnp_Ra_c19079_28210937*	3A	123.35	AA	47.85		0.02^*^					
	*wsnp_Ex_c12341_19693570*	3A	127.51	CC	57.21	0.14^**^			0.20^**^		0.21^**^	
	*wsnp_Ku_c29102_39008953*	3B	123.29	CC	25.96			0.10^*^				
	*wsnp_Ex_c13154_20785032*	3B	127.87	GG	23.44	0.11^*^						
	*wsnp_Ex_rep_c67136_65617520*	4B	108.15	CC	87.25			0.05^*^				
	*wsnp_JD_rep_c49046_33288885*	5A	116.00	CC	92.56			0.31^**^				0.15^**^
	*wsnp_Ku_c14275_22535693*	5A	116.57	AA	90.70				0.15^*^			
	*wsnp_Ku_c14275_22535576*	5A	116.83	TT	90.70						0.17^*^	
	*wsnp_Ex_c43578_49857984*	5A	130.98	TT	72.56		0.20^**^					0.20^**^
	*wsnp_Ex_rep_c101757_87064771*	5A	133.01	CC	97.21			0.18^*^				
	*wsnp_Ex_rep_c101757_87065169*	5A	133.17	CC	90.70	0.18^*^						
	*wsnp_Ex_c57667_59284398*	5B	125.89	CC	56.59	0.07^*^						
	*wsnp_Ex_c1050_2009301*	6A	45.73	TT	85.10				0.19^*^			
	*wsnp_Ex_c19467_28423197*	6B	50.70	AA	7.62	0.26^*^						
	*wsnp_Ex_c65899_64135487*	7D	1.12	AA	95.12		0.01^*^					
TKW	*wsnp_Ex_c3685_6723631*	2A	9.91	GG	34.11							1.07^*^
	*wsnp_BE406351A_Ta_2_2*	2A	112.62	TT	66.03	0.83^*^						
	*wsnp_RFL_Contig2914_2757372*	2B	211.84	AA	61.46					1.19^*^		
	*wsnp_Ku_rep_c69511_68887456*	3A	59.73	TT	55.77	1.41^**^	1.01^**^	1.48^**^				1.33^**^
	*wsnp_CAP12_c4769_2174195*	4B	106.45	CC	89.76				1.26^*^			
	*wsnp_BF291478B_Ta_2_1*	6B	80.00	CC	84.21	1.18^*^						

^*^indicates significant at *P* < 0.05 ^**^indicates significant at *P* < 0.01

### Transmission of favorable alleles from founder parents

**Fig. 3:**
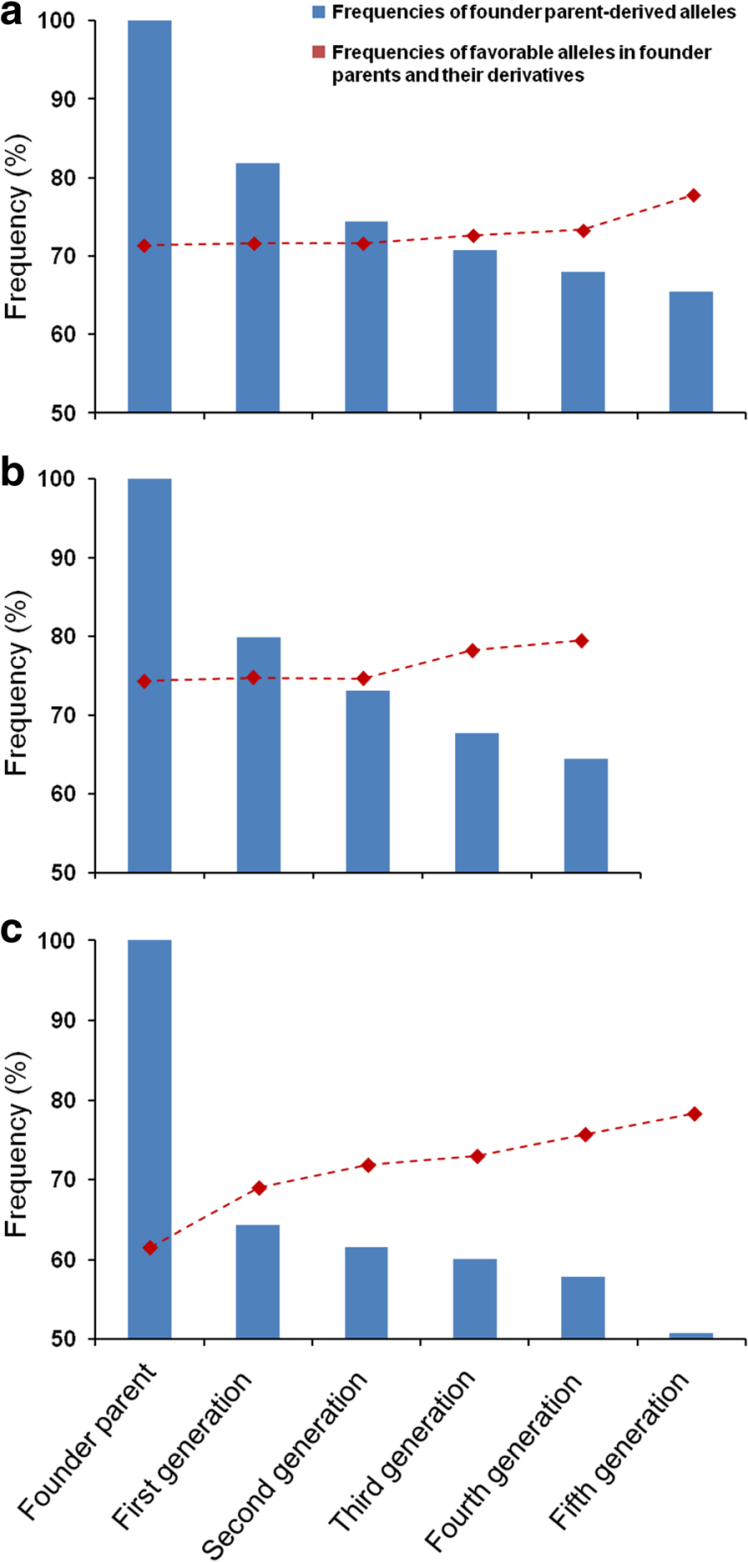
Frequencies of favorable alleles and founder parent-derived alleles in three founder parents and their derivatives. Blue bars represent the frequencies of founder parent-derived alleles and the red dotted lines indicate the frequencies of favorable alleles in founder parents and their derivatives. **a** Funo; **b** Bima 4; **c** Nanda 2419

All 76 alleles with a positive effect on yield-related traits identified in the association analysis were used to analyze the transmission rates of alleles from founder parents to progenies, as well as the frequencies of favorable alleles in later generations. Transmission rates from the first generation of Funo to the fifth generation were between 81.88 and 65.48%, and the frequencies of favorable alleles in different generations changed from 71.99 to 78.21%. Transmission rates from the first generation of Bima 4 to the fourth generation were between 79.94 and 64.38%, and frequencies of favorable alleles increased from 74.79 to 79.49%. Likewise, transmission rates for first to fifth generation derivatives of Nanda 2419 were between 64.25 and 50.72%, while the corresponding frequencies of favorable alleles increased from 68.91 to 78.21% (Fig. 3). Although the transmission rates of alleles from founder parents decreased with the number of generations, the percentage of favorable alleles increased.

**Fig. 4:**
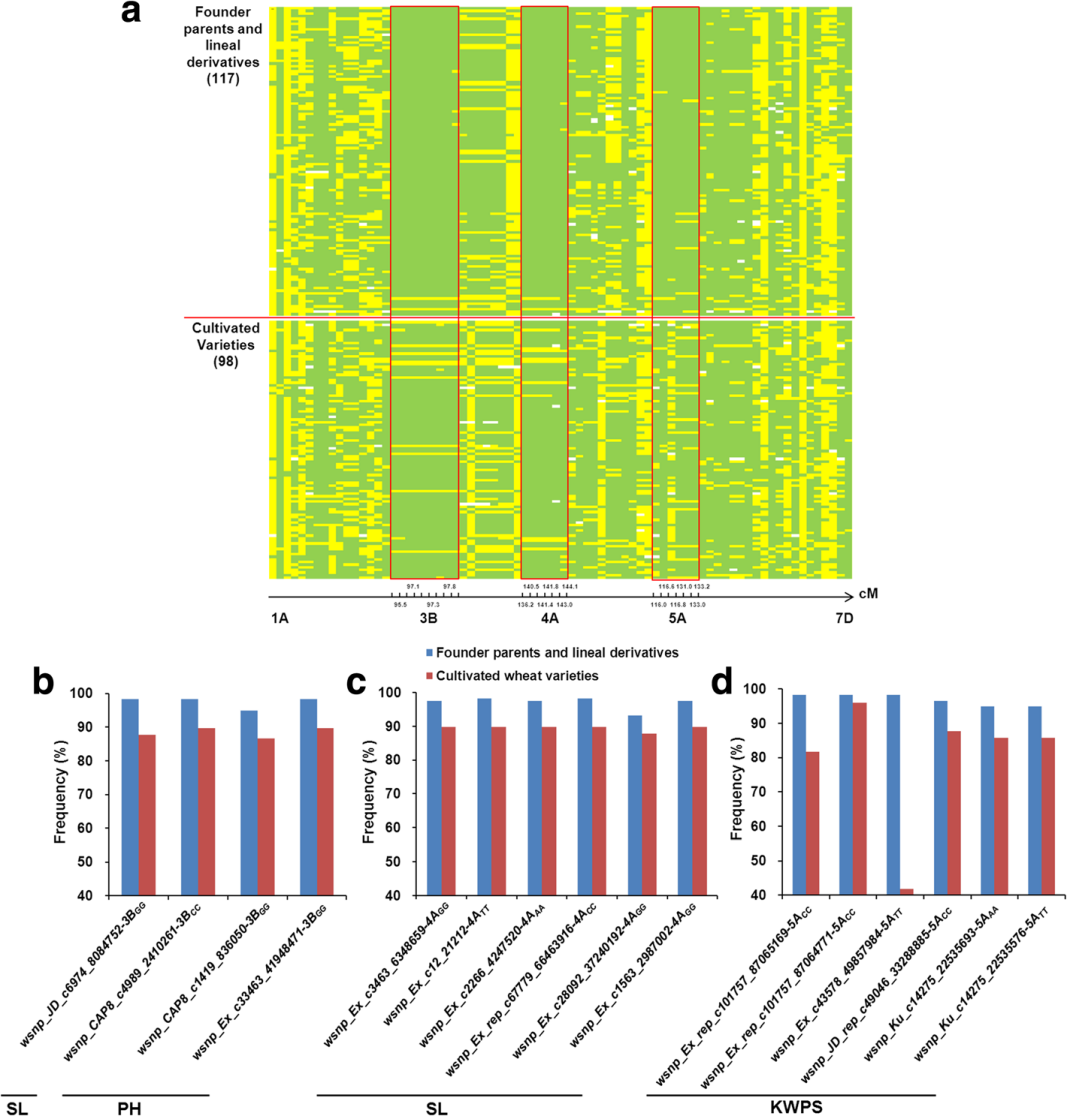
Distributions of favorable alleles associated with agronomic traits in 215 cultivated varieties. Green indicates the favored allele at each locus, yellow indicates alternative alleles, white indicates missing data. **a** comparative distribution of favorable alleles associated with agronomic traits in 117 founder parents and derivatives and 98 independent varieties; **b** frequencies of favorable alleles associated with SL and PH in the chromosome 3B region 95.5–97.8 cM in the founder parents and derivatives and other cultivated varieties; **c** frequencies of favorable alleles associated with SL in the 4A region 136.2–144.1 cM after comparing founder parents and derivatives with an independent variety group; **d** frequencies of favorable alleles associated with KWPS in 5A region 116.0–133.2 cM after comparing founder parents and derivatives with cultivated varieties

**Fig. 5:**
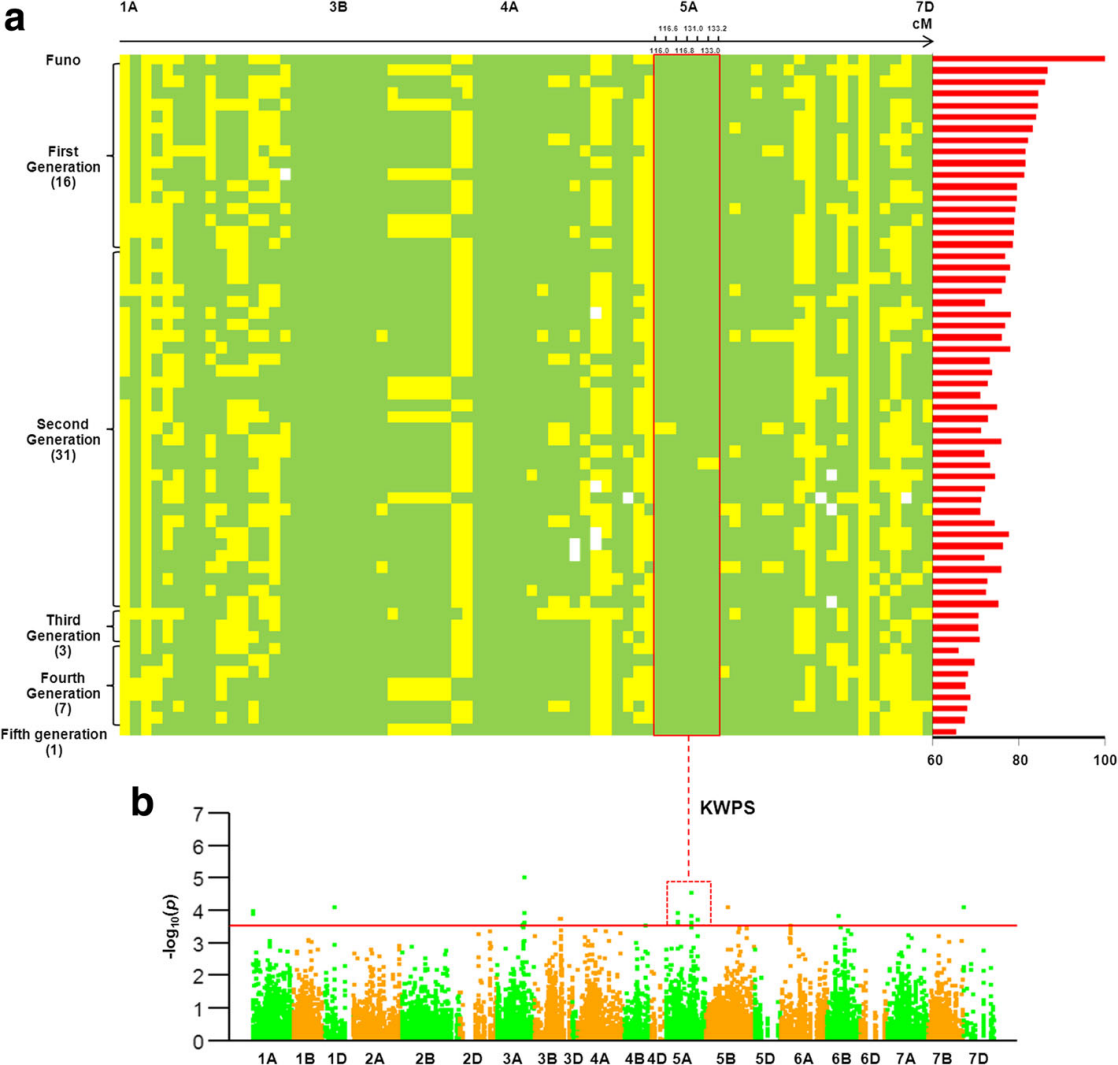
Distribution of favorable alleles associated with agronomic traits in Funo and its derivatives. Green indicates the favorable allele at each locus, yellow indicates the alternative allele, white indicates missing data, red histogram represents the frequencies of founder parent-derived alleles. (**a**) distribution of favorable alleles associated with agronomic traits in Funo and its derivatives; (**b**) Manhattan plot displaying the GWAS result for KWPS with 3792 SNPs in six environments

Overall analysis of chromosome regions involving 76 favorable alleles showed that among the 15 chromosomes with association signals for agricultural traits, only three regions, 95.5–97.8 cM on 3B, 136.2–144.1 cM on 4A, and 116.0–133.2 cM on 5A had high frequencies for alleles with a positive influence on yield traits (Fig. 4a). In particular, the 3B region was associated with SL and PH (Fig. 4b), while the 4A region associated with SL (Fig. 4c). Additionally, the 116.0–133.2 cM region on 5A was present in derivate cultivars with high frequency and associated with KWPS (Figs. 4d and 5).

## Discussion

### Genetic diversity of founder parents and derivatives

One hundred and seventeen of the 215 cultivars investigated in this study were first to fifth generation derivatives of Funo, Nanda 2419 and Bima 4 that were bred in different provinces of China. The 215 cultivars were divided into two groups, first (162 accessions, 75.3%) including Funo, Nanda 2419, and their derived offspring, while the second (53, 24.7%) included Bima 4 and many of its derived offspring. Pedigree analysis showed that the first generation derivatives of Funo Sumai 2 and Sumai 3, as well as the second generation Ning’ai 8628 and Wu 7815–4-1, clustered together. Moreover, the first generation of Funo derivatives obtained from a cross with Neixiang 5 (Zhengzhou 17, Kaifeng 10 and Xuchang 26), and the second generation obtained from crosses involving Zhengzhou 17 (Sudi 8112, Zhengzhou 741, Huapei 128–8 and Xiangmai 5) were also in the same cluster. Thus, different cultivars from the same original cross had high similarity, indicating little genetic differences in the traits analyzed. Moreover, among different clusters, genetically related lines mostly grouped in the same cluster, indicating the results were consistent with the genealogy (Additional file 4: Table S4). However, a few lines with direct pedigree relationship to a particular founder did not fall into the same group. For instance, 16 of the 17 first generation Funo lines belonged to the first group, but Linnong 14, which are 50% related to Funo, fell into the second group, showing that high performance offspring with large differences could be selectively bred from the same founder parent.

### Dissection of founder parents by favorable alleles

Previous studies found that the genes controlling important traits tended to be clustered rather than randomly distributed on chromosomes [24–27]. For example, Huang et al. [25] identified QTLs for TKW and KNPS in the *Xgwm334a-Xgwm1043* region on chromosome 6A, PH, KNPS, and TKW near *Xgwm786* on chromosome 7D, and KNPS, spike weight, heading date, TKW, and PH in the *Xgwm1220-Xgwm1002* region also on chromosome 7D. Li et al. [26] localized eight QTLs for TKW, spike number per square meter, sterile spikelet number per spike and fertile spikelet number per spike near markers *Xwmc31*, *Xgdm67*, and *Xgwm428* on chromosome 7D.

We investigated favorable allele combinations carried by the founder parents and found that among the 76 associated loci, Bima 4, Funo, and Nanda 2419 carried 58, 56 and 48 favorable alleles, respectively. Among the 25 associated loci detected in multiple environments, Bima 4, Funo and Nanda 2419 carried 20, 19 and 14 favorable alleles, respectively. In particular, the *wsnp_CAP8_c1419_836050* - *wsnp_Ex_c6129_10723019* region on chromosome 3B associated with both SL and KNPS; and *wsnp_Ex_c1563_2987002* - *wsnp_Ex_c3463_6348659* on chromosome 4A associated with SL. Favorable alleles in these two segments were present with high frequency in Bima 4, Funo, and Nanda 2419, indicating that these varieties have potential for breeding programs. Similarly, the *wsnp_Ku_c29102_39008953* – *wsnp_Ex_c13154_20785032* region of chromosome 3B associated with KNPS. This segment was linked to yield increase in both Bima 4 and Funo.

### Implications for molecular wheat breeding

Shoemaker et al. [28] suggested that the process of plant breeding reflects how breeders “manipulate” traits to preferentially select for high yield, disease resistance, and high quality. In this study, 27 of 76 marker-trait associations (MTAs) co-localized with previously identified QTL or loci (Additional file 5: Table S5) [7, 13, 28–32]. SNPs *wsnp_Ku_c29102_39008953*, *wsnp_Ex_c11837_18996495* and *wsnp_Ex_c12781_20280445* located in region 123.3–126.6 cM on chromosome 3B affected KWPS, while genes for KNPS near locus *BS00022025_51* associated with TKW [13]. *QSL.caas-4AS* for SL mapped to region 140.5–144.1 cM, which included loci *wsnp_Ex_c28092_37240192*, *wsnp_Ex_rep_c67779_66463916*, *wsnp_Ex_c12_21212*, and *wsnp_Ex_c3463_6348659* identified in the current GWAS [7]. We found that favorable alleles associated with spike weight in founder parents and derivatives were clustered in the 116.0–133.2 cM region on chromosome 5A (Figs. 3 and 4). Gao et al. [7] also indicated that QTLs *QNDVI-A.caas-5AL*, *QChl-A.caas-5AL* and *QChl-10.caas-5AL* in this region might affect yield.

Twenty-five SNP loci associated with yield-related traits in two or more of six environments. Among them, eight SNP loci co-localized with those found in previous studies (Table 2 and Additional file 5: Table S5) [7, 13, 28–32]. Thus, these favorable alleles, especially locus *wsnp_Ex_c49211_53875575-5A* detected in all six environments, is interesting for future breeding programs.

## Conclusions

Two hundred and fifteen wheat cultivars were genotyped by the 9 K SNP iSelect assay and all were phenotyped for six yield-related traits in six environments. Comparisons of yield-related traits in founder parents Funo, Bima 4, Nanda 2419, and their derivatives indicated that breeders applied a strong selective pressure on PH and TKW. MAF, PIC and gene diversity analysis using 3792 SNP markers showed high genetic diversity. Genome-wide association analysis of yield-related traits detected 117 significant associations at 76 SNP loci on 15 chromosomes. Twenty five associations were detected in two or more environments. Three regions with high-frequencies of favorable alleles were identified in position 95.5–97.8 cM on chromosome 3B, and in position 136.2–144.1 cM and 116.0–133.2 cM on chromosome 5A. The region on chromosome 5A associated with KWPS was highly distinctive in favorable alleles between founder and derived lines compared to other cultivars. Our findings partially identify the genetic basis of the role of founder parents in crop breeding, and provide information for future wheat improvement by marker-assisted selection.

## Methods

### Plant materials

The plant material was a collection of 215 wheat cultivars, including 11 founder parents and 106 derivatives and 98 other varieties (Additional file 4: Table S4). The first group comprised 11 founder parents, such as Funo, Bima 4 and Nanda 2419 (Additional file 6: Figure S1) [33], and they have made significant contributions to Chinese wheat breeding and 106 derivatives of those parents. The other 98 genotypes originated from Italy (2) and Chinese provinces including Anhui (4), Beijing (5), Fujian (5), Gansu (2), Guizhou (1), Hebei (4), Henan (9), Hubei (3), Hunan (8), Jiangsu (16), Jiangxi (1), Shaanxi (17), Shandong (12), Shanxi (3) and Sichuan (6). Details are provided in Additional file 4: Table S4.

### Phenotyping

The whole germplasm set was planted at three locations (Taian in Shandong; Yangling in Shaanxi; and Yangzhou in Jiangsu) in two growing seasons (2008–2009 and 2009–2010). Field management followed local practices. The six irrigated environments were designated 09TA, 10TA, 09YL, 10YL, 09YZ and 10YZ. Field experiments were grown in randomized block designs with three replications. Each line was planted in 2 m, 5 row plots at 40 kernels per row, with a row spacing of 20 cm. The agronomic traits PH and TKW were measured at maturity. Thirty spikes of each line were randomly collected from the middle row and used for measurements of SL, SNPS, KNPS, and KWPS.

### Genotyping and statistical analysis

Genomic DNA extraction was carried out using the CTAB method [34]. Descriptive statistical analysis and analysis of variance (ANOVA) of phenotypic data were calculated by using SPSS 21.0 (http://www.brothersoft.com/ibm-spss-statistics-469577.html). The best linear unbiased prediction (BLUP) method was used to calculate the mean values of each trait [35–37].

SNP genotyping was performed on the BeadStation and iScan instruments and conducted at the Genome Center of the University of California at Davis according to the manufacturer’s protocols (Illumina, USA) [5]. Data correction, input and output performed using GenomeStudio v2011.1 [38]. Information on chromosome location of polymorphic SNPs was obtained from Cavanagh et al. [5]. PowerMarker V3.25 was used to estimate genetic diversity of SNPs [39]. Population structure of the 215 cultivars was evaluated with 3792 SNP markers distributed on all 21 chromosomes using Structure 2.3.4 with a burn-in period at 50,000 iterations and a run of 500,000 replications of Markov Chain Monte Carlo (MCMC) after burn in [40]. For each run, 5 independent runs were performed with the number of cluster K varying from 1 to 10, leading to 50 Structure outputs. Then the number of populations was estimated on the basis of the Evanno criterion [41]. Based on the Q + K model [42, 43] and TASSEL 5.0 software [31] (http://www.maizegenetics.net), GWAS was performed using the yield-related traits and SNP marker data. After exclusion of SNP loci with frequencies < 0.05, a uniform suggestive genome-wide significance threshold (1/3271 = 3.06 × 10^− 4^, or *P* < 3.06 × 10^− 4^, -Log*P* > 3.51) was given.

The 215 wheat cultivars were grouped by the neighbor-joining method in MEGA 5.0 [32]. The transmission frequencies of alleles from founder parents to later generations as well as favorable alleles were computed in this study. The transmission rate was defined as the percentage of average numbers of alleles carried by one generation derived from the founder parent relative to the total number of alleles detected. The frequency of favorable alleles was defined as the percentage of average numbers of favorable alleles carried by one generation relative to the total number of favorable alleles detected.

## Additional files

Additional file 1: Table S1. Pearson’s correlation coefficients between phenotypic traits in different environments. (XLSX 10 kb)

Additional file 2: Table S2. Descriptive statistics (Means ± SD) of six phenotypic traits in three founder parents and their derivatives in different environments. (XLSX 20 kb)

Additional file 3: Table S3. Allelic number, MAF and PIC of 3792 SNP markers detected in this study. (XLSX 304 kb)

Additional file 4: Table S4. Information for 215 wheat accessions used in this study. (XLSX 24 kb)

Additional file 5: Table S5. Significant MTAs identified in current and previous study. (XLSX 13 kb)

Additional file 6: Figure S1. The pedigree sketch of wheat varieties cultivated in large scale and their founder genotypes. (TIFF 6380 kb)

## Abbreviations

GWAS: Genome-wide association studies
KNPS: Kernel number per spike
KWPS: Kernel weight per spike
MAS: Marker assisted selection
MTAs: Marker-trait associations
PH: Plant height
SL: Spike length
SNP: Single nucleotide polymorphism
SNPS: Spikelet number per spike
TKW: Thousand kernel weight
