# Supplementary figures and images for: Association of yield-related traits in founder genotypes and derivatives of common wheat (Triticum aestivum L.)
Source: BMC Plant Biol. 2018 Feb 20;18:38. doi: 10.1186/s12870-018-1234-4 (PMC5819277; doi:10.1186/s12870-018-1234-4)

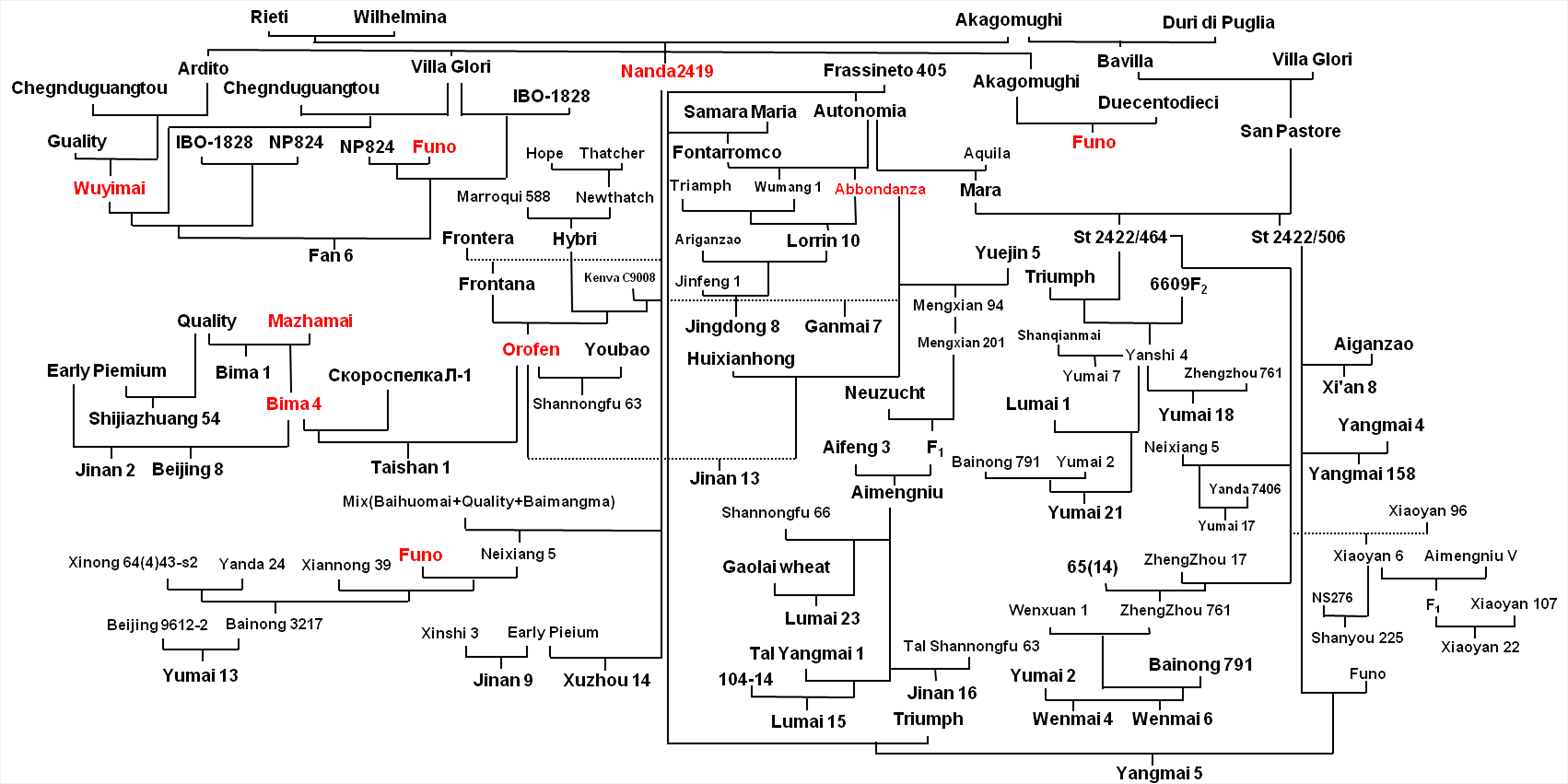

Supplement: Supplementary file 6 — The pedigree sketch of wheat varieties cultivated in large scale and their founder genotypes. (TIFF 6380 kb) [file 12870_2018_1234_MOESM6_ESM.tif]
